# Prevalence and occupational risk factors of musculoskeletal diseases and pain among dental professionals in Western countries: A systematic literature review and meta-analysis

**DOI:** 10.1371/journal.pone.0208628

**Published:** 2018-12-18

**Authors:** Janna Lietz, Agnessa Kozak, Albert Nienhaus

**Affiliations:** 1 Competence Center for Epidemiology and Health Services Research for Healthcare Professionals (CVcare), University Medical Center Hamburg-Eppendorf (UKE), Hamburg, Germany; 2 Department of Occupational Medicine, Hazardous Substances and Public Health (AGG), Institution for Statutory Accident Insurance and Prevention in the Health and Welfare Services (BGW), Hamburg, Germany; James Cook University, AUSTRALIA

## Abstract

**Background:**

This review aimed at examining the prevalence of musculoskeletal diseases and pain among dental professionals in Western countries. Furthermore, possible occupational risk factors were analyzed.

**Methods:**

The literature search was conducted from June to July 2016, with an update in December 2017 using the databases MEDLINE, CINAHL, LIVIVO, Science Direct, PubMed, and Web of Science. The quality assessment was performed with a standardized instrument consisting of 10 items. A meta-analysis was carried out to compute pooled prevalence rates for musculoskeletal diseases and pain.

**Results:**

A total of 41 studies were included in this review; 30 studies met the criteria for the meta-analysis. Prevalence rates of musculoskeletal diseases and pain among dental professionals ranged from 10.8% to 97.9%. The neck was the body region affected most often (58.5%, 95% CI = 46.0–71.0) followed by the lower back (56.4%, 95% CI = 46.1–66.8), the shoulder (43.1%, 95% CI = 30.7–55.5) and the upper back (41.1%, 95% CI = 32.3–49.9). Potential occupational risk factors included an awkward working posture, high number of treated patients, administrative work, vibration, and repetition.

**Conclusions:**

Musculoskeletal diseases and pain are a significant health burden for dental professionals. This study showed high prevalence rates for several body regions. Therefore, suitable interventions for preventing musculoskeletal diseases and pain among dental professionals are needed.

## Background

Musculoskeletal diseases and pain are a major health concern among dental professionals in Western countries. Musculoskeletal diseases are defined as a group of diseases and complaints that affect different structures of the musculoskeletal system. These include the nerves, tendons, muscles, joints, ligaments, bones, blood vessels, and supporting structures such as intervertebral discs [1–3]. Musculoskeletal diseases and pain can occur from a single or cumulative trauma and cause pain or sensory disturbances in various regions of the body like the back, neck or shoulders. They can develop either as acute or chronic conditions—the latter are common, representing 30 to 40% of all chronic diseases [2,3]. Some studies have reported that musculoskeletal diseases and pain considerably contribute to reduced productivity and poorer quality of work, decreased job satisfaction, occupational accidents, sick leave, and leaving the profession via premature retirement. Moreover, musculoskeletal diseases and pain can result in high expenditures for medical treatments [1,2,4,5]. In Germany, for instance, the medical costs for musculoskeletal diseases and pain amounted to 34.2 billion euros in 2015, equaling 10.1% of all medical expenditures [6].

Having a healthy musculoskeletal system is especially important for dental professionals, as dentistry is a physically and mentally demanding occupation. In their work, dental professionals have to perform precise hand movements, use vibrating instruments, adopt static postures, use psychomotor skills, and perform repetitive monotonous tasks over long periods of time [7]. As a result, it is necessary to have a deep understanding of the development and the occupational etiology of musculoskeletal diseases and pain among dental professionals. Musculoskeletal diseases and pain are influenced by various factors, including physical (e.g., height, weight, sex), occupational (e.g., overuse of a body region, uncomfortable posture, insufficient breaks), and socio-psychological characteristics (e.g., high work intensity, stress) [8].

There are some systematic literature reviews focusing on the morbidity and etiology of musculoskeletal diseases and pain among dental professionals. The last one was published by Hayes et al. in 2009 [9]. Therefore, a systematic review and assessment of current studies is warranted.

The objective of the current study was to determine the frequency and severity of musculoskeletal diseases and pain among dental professionals in Western countries. The focus lay on analyzing the prevalence in different regions of the body. Moreover, studies on possible occupational risk factors for musculoskeletal diseases and pain were reviewed.

## Methods

The present review was performed systematically in line with the proposal for reporting Meta-analyses Of Observational Studies in Epidemiology (MOOSE checklist) [10].

The study protocol for this review was written in accordance with the Preferred Reporting Items for Systematic review and Meta-Analysis Protocols (PRISMA-P) statement [11]. It can be obtained from the corresponding author. The protocol is available in English and documents intended research methods for this review process.

Neither an ethics committee approval nor informed consent were required for the systematic review of published literature. There was no contact to possible study participants at any time.

### Eligibility criteria

For the screening and eligibility assessment of identified sources, several criteria were defined in line with the PEOS (population, exposure, outcome, study design) criteria. Studies were included if the study population consisted of dental professionals working in general oral healthcare facilities. Dental professionals comprised, for example: dentists, orthodontists, dental surgeons / hygienists / assistants / technicians, and dental students. The study population was exposed to specific working conditions facing dentistry. The exposure was therefore represented by the occupation in dentistry. Moreover, studies were considered if they analyzed prevalence rates and/or occupational risk factors. Outcome measures were musculoskeletal diseases and/or pain. Musculoskeletal diseases (e.g., carpal tunnel syndrome, median mononeuropathy, or osteoarthritis) can be diagnosed by validated and standardized diagnostic methods like laboratory tests based on blood samples, radiographs, computed tomography (CT) scans, magnetic resonance imaging (MRI), bone scanning, arthroscopy and ultrasonography [12,13]. Musculoskeletal pain (e.g., back, neck, and shoulder pain or hand symptoms) is often nonspecific and thus more difficult to be verified. Clinical provocation tests, nerve conduction/muscle tests, sensation tests, and palpation can be used to verify musculoskeletal pain reported by patients [14,15]. All types of pain (e.g., acute vs. chronic, specific vs. nonspecific) were considered. Only observational studies such as cross-sectional studies (prevalence studies), retrospective and prospective cohort studies, and case-control studies were included. The studies had to be published in peer-reviewed journals and accessible as full-text. Appropriate study settings were dental practices, orthodontic practices, dental clinics / hospitals, and dental schools. The authors only selected studies published in English.

### Filter criteria

In addition to the eligibility criteria some filter criteria were defined during the later review process. After the full-text screening and following eligibility assessment, 179 studies were considered suitable for inclusion based on the predefined eligibility criteria. Especially due to the large number of studies, further exclusion criteria were applied in stages. Firstly, studies published before 2005 were excluded. Secondly, the authors excluded all studies that contained fewer than 50 study participants. Thirdly, studies with less than 50% of quality criteria (see below) met were not considered. Finally, all studies carried out in Africa, Asia, and Central and South America were excluded. Further reasons for selecting these filter criteria were as follows: a) we excluded studies before 2005 for being able to display a current state of knowledge on this topic. Hence we decided to analyze studies not older than 13 years. b) We did not consider studies with fewer than 50 participants as the empirical validity is assumed to be limited in smaller studies. c) We excluded studies from developing countries (Africa, Asia, Central and South America) as working conditions of dental professionals and occupational safety differ significantly from those in industrialized countries. The comparability of study results would not be given.

### Information sources and search strategy

The systematic database search was carried out from June to July 2016 in MEDLINE, CINAHL, LIVIVO, Science Direct, PubMed, and Web of Science. Moreover, reference lists of included studies and relevant review articles were hand searched to uncover further sources. Other scientific experts of this study topic were contacted by email to receive additional information about current publications.

The following search terms and Medical Subject Headings (MeSH) were used to search all included databases, for instance:

- Dent* OR dental personnel OR oral health OR orthodontists

AND

- Occupational exposure OR occupational risk* OR risk factors

AND

- Musculoskeletal diseases OR musculoskeletal pain OR work-related upper-extremity musculoskeletal disorders.

A detailed description of the general search strategy is provided in **S1 Appendix**. The strategy was adapted to the setup of the individual database.

A systematic update search in December 2017 revealed 14 further studies fulfilling the eligibility criteria of this review. After the consideration of all filter criteria, 2 studies remained [16,17].

### Literature screening

The literature screening and eligibility assessment of the studies were performed independently by two authors (JL and AK). The screening process comprised title and abstract screening as well as full-text screening. For this purpose, a standardized screening instrument was developed. If a study met all specified eligibility and filter criteria, it was included in the review. Disagreements between the two authors were resolved by discussion. JL and AK ultimately agreed on all included studies.

### Data collection

Data extraction for the included studies was carried out by JL and AK independently. A standardized data extraction tool was developed for collecting information on study characteristics (e.g., study design, location, setting, study population) and study results (e.g., sample size, prevalence rates, risk estimates). The extraction tool consisted of 20 items. In case of uncertainty, a discussion took place between the authors. JL extracted the detailed data using Microsoft Excel 2013 spreadsheets. If possible, a calculation of missing values was performed. Some study authors were contacted by email to obtain more information on the presented results or missing data.

### Quality assessment

Following the screening and data extraction, the included studies were assessed in terms of study quality. The assessment was performed by two authors (JL and AK) independently. Diverging results were discussed and resolved among the authors. A standardized instrument was created for this quality assessment. It comprised 10 items that were categorized in 6 quality criteria (**Table 1**). All items were taken from two well-validated checklists [18–20] and modified. The items (e.g., “the study population was clearly described”) were to be answered with “yes” (1 point), “no” (0 points), or “unclear” (0 points). The study quality was finally assessed by adding up the points. This yielded a scale from 0 to 10. Only studies with at least five points were included. Studies with a score from 10 to 8 were considered of high quality and studies with a score from 7 to 5 of moderate quality.

**Table 1:** Checklist for the quality assessment of studies analyzing MSDs/MSP among dental professionals.

Number	Criterion	Item
1	Study purpose	A specific, clearly stated purpose of the study was described.*
2	Study design	A prospective/retrospective design was used.**
3	Study population	The study population was clearly described.*
4		The participation rate was ≥ 70% (baseline).**
5	Assessment of exposure	The occupational exposure was clearly defined.**
6		The exposure was assessed by a standardized method.**
7	Assessment of outcome	The outcome was clearly defined.**
8		The outcome was assessed by a standardized method.**
9	Analysis and data presentation	Risk estimates or raw data were given.*
10		The study controlled for confounding.*

* Item adapted from [18,19].

** Item adapted from [20].

### Statistical analysis and data synthesis

A meta-analysis was conducted to enable comparability of prevalence data from different studies. Data on prevalence of musculoskeletal diseases and pain were extracted and pooled using the Microsoft Excel spreadsheet developed by Neyeloff et al. [21]. Pooled prevalence rates were calculated separately by the prevalence period for the total prevalence and for prevalence in different body regions. Only studies that used the following prevalence periods were considered: point (current), weekly (7 days) and annual (12 months) prevalence. Heterogeneity was quantified using the I^2^ statistics with I^2^ ≥ 25% considering low, ≥ 50% moderate and ≥ 75% high heterogeneity [22]. If heterogeneity of the effects between studies existed, random- effects models were used to compute the pooled effect estimate for the prevalence of musculoskeletal diseases and pain. The pooled prevalence rates were reported with 95% confidence intervals.

Depending on availability, we report odds ratios (OR) or adjusted OR (AOR) from studies analyzing risk factors for musculoskeletal diseases and pain. No meta-analysis was performed for this part, as the exposures and outcomes were not homogenously defined.

## Results

### Study selection

**Fig 1:**
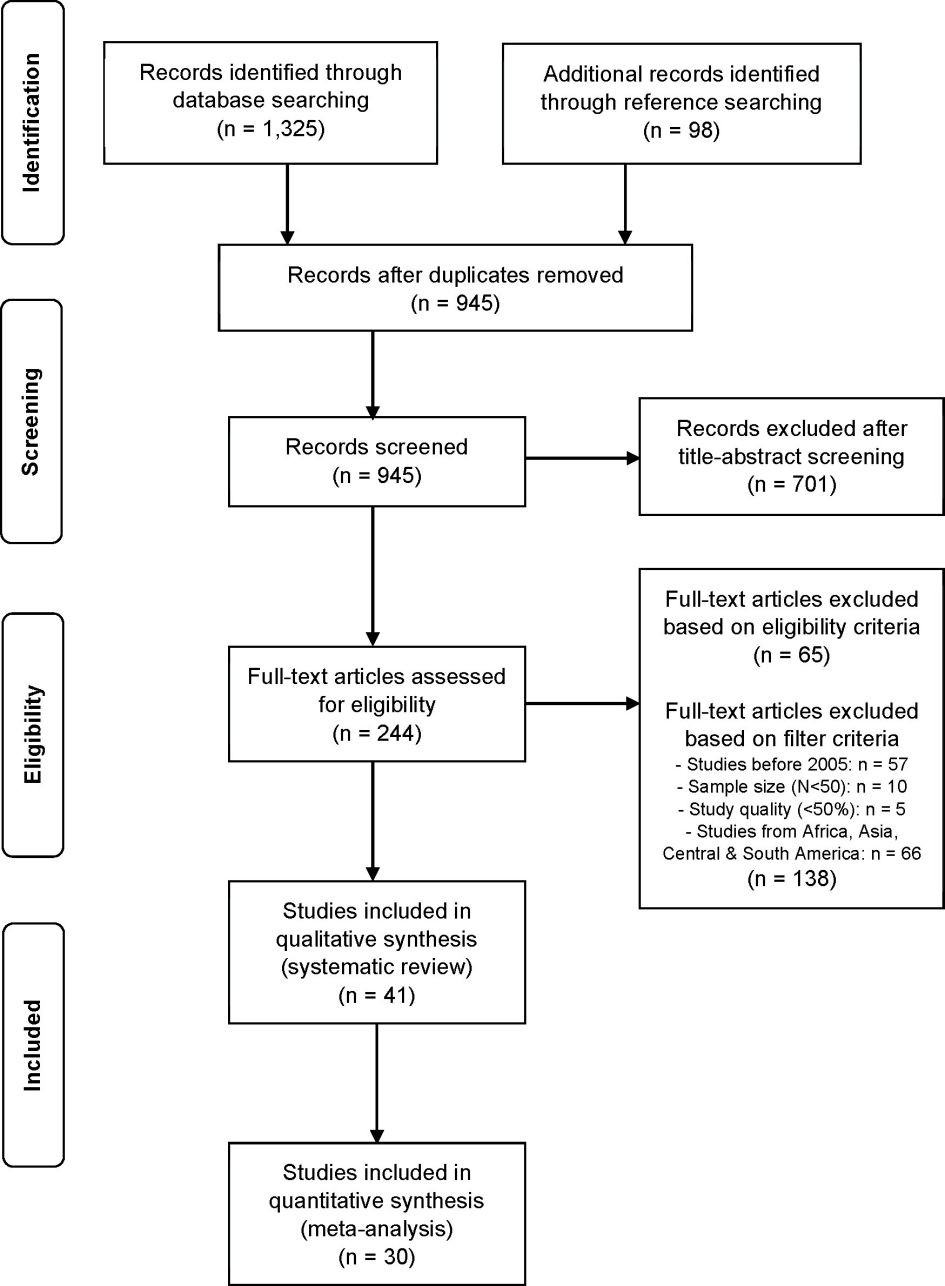
Study selection process for this systematic review (PRISMA flowchart).

The literature search yielded 1,325 titles (**Fig 1**). Through reference searching, 98 additional studies were found. After adjusting for duplicates, 945 titles remained. Of these, 701 studies were excluded after the title- and abstract screening, as they did not fulfill the eligibility criteria. Of the remaining 244 studies that were subjected to the full-text screening, 65 did not meet the eligibility criteria. Based on the additional filter criteria, 138 sources were excluded afterwards. Main reasons for exclusion from this review were a different study topic (e.g., interventions related to MSDs/MSP), study population (e.g., patients) or a divergent study design (e.g., review, intervention study). In the end, 41 studies were considered suitable to be included in this review. They comprised 33 cross-sectional studies, 3 cohort studies and 5 case-control studies. Of these, 30 studies (73.1%) met the criteria for the meta-analysis. Reasons for exclusion of 11 studies [17,23–32] from the meta-analysis were a differing prevalence period or missing prevalence data.

### Study characteristics

All included sources were observational studies analyzing prevalence rates and/or occupational risk factors of musculoskeletal diseases and pain among dental professionals. They were published in English between 2005 and 2017, with the most studies issued per year in 2006 (n = 7; **Table 2**). In line with the fourth filter criterion, all studies were conducted in Western countries. Approximately half of the studies (n = 23) came from Europe (e.g., Sweden, Finland, Croatia, and Spain) and one quarter each from North America (USA; n = 10) and Oceania (Australia and New Zealand; n = 8). Seventeen studies (41.4%) took place in general dental practices, 16 (39.0%) in dental hospitals/clinics, 15 (36.5%) in dental schools, 5 (12.1%) in orthodontic practices, and one study (2.4%) in an endodontic practice. Some studies were carried out in multiple settings.

**Table 2:** Study characteristics of included studies analyzing MSDs/MSP among dental professionals (n = 41).

Reference	Country	Setting	Study population	Sample size (N)	Dental profes-sionals (n)	Prevalence: period of time^a^	Prevalence: study outcome	Prevalence of MSDs/MSP (n+, %)^b^^,^^c^	Occupational risk factors	Study quality score
Cross-sectional studies (n = 33)										
Abou-Atme 2006 [33]	Italy, Europe & Lebanon, Asia	dental school, university	dental students, non-dental students	530	292	current	a) TMJ pain; b) TMJ sounds	a) Italian dental students: 21 (18.4), Lebanese dental students: 25 (14.0); b) Italian dental students: 38 (33.3), Lebanese dental students: 48 (27.0)	not surveyed	5 (moderate)
Ayers 2009 [7]	New Zealand, Oceania	dental practice	dentists	566	566	7 days, 12 months	MSP (general)	see Table 3	not surveyed	7 (moderate)
Cherniack 2006 [51]	USA, North America	dental hospital/clinic, dental school	dental hygienists, dental hygiene students	160	160	12 months	a) MSP; b) CTS	a) 108 (67.5); b) 20 (12.5)	vibration	8 (high)
Ding 2007 [25]	Finland, Europe	dental practice, schools	dentists, teachers	543	295	1 month	osteoarthritis	32 (10.8)	not surveyed	7 (moderate)
Gijbels 2006 [14]	Belgium, Europe	dental practice, dental hospital/clinic	dentists	388	388	current	MSDs (general)	43 (11.0)	not surveyed	7 (moderate)
Harutunian 2011 [23]	Spain, Europe	dental school	teaching faculty members, dental students	74	74	6 months	MSDs (general)	59 (79.7)	not surveyed	8 (high)
Hayes 2009a [34]	Australia, Oceania	dental school	dental hygiene students	126	126	12 months	MSP (general)	see Table 3	administrative work	9 (high)
Hayes 2012 [27]	Australia, Oceania	dental practice, orthodontic practice	dental hygienists	560	560	not stated	MSDs (general)	not stated	workplace, hand scaling, sonic/ ultrasonic scaling	7 (moderate)
Hayes 2013 [35]	Australia, Oceania	dental practice, orthodontic practice	dental hygienists	560	560	12 months	MSP (general)	see Table 3	not surveyed	7 (moderate)
Hodacova 2015 [36]	Czech Republic, Europe	dental practice, orthodontic practice, dental hospital/clinic	dentists	575	575	12 months	MSDs (general)	563 (97.9)	workload/job demand, number of treated patients	9 (high)
Humann 2015 [37]	USA, North America	dental hospital/clinic	dental hygienists	488	488	current	MSDs (general)	468 (95.9)	not surveyed	7 (moderate)
Kierklo 2011 [38]	Poland, Europe	dental practice, orthodontic practice, dental hospital/clinic	dentists	220	220	current	MSDs (general)	202 (91.8)	not surveyed	5 (moderate)
Kulcu 2010 [39]	West Turkey, Europe	dental school	dentists, dental nurses, dental students	206	206	current	a) low back pain; b) neck pain	a) 125 (60.6); b) 70 (33.9)	not surveyed	6 (moderate)
Leggat 2006 [40]	Australia, Oceania	dental practice, dental hospital/clinic	dentists	285	285	12 months	MSDs (general)	249 (87.3)	not surveyed	8 (high)
Lindfors 2006 [41]	Sweden, Europe	dental hospital/clinic	dental professionals (dentists, dental hygienists, dental nurses)	945	945	current	MSDs (general)	765 (80.9)	not surveyed	9 (high)
Nordander 2013 [57]	Sweden, Europe	dental practice, dental hospital/clinic	dentists, dental hygienists	not stated	84	7 days, 12 months	MSDs (general)	55 (65.5), 81 (96.4)	not surveyed	6 (moderate)
Palliser 2005 [42]	New Zealand, Oceania	dental practice, dental hospital/clinic	dentists	413	413	7 days, 12 months	MSP (general)	see Table 3	not surveyed	9 (high)
Pejcic 2017 [16]	Serbia, Europe	dental practice, orthodontic practice, dental hospital/clinic	dental professionals (dentists, oral surgery specialists, endodontists, orthodontists, prosthodontics, general dental consultants, pediatric dental consultants)	356	356	current	a) MSDs (general); b) CTS	a) 294 (82.5); b) 81 (22.8)	working time (hours/days), no breaks, number of treated patients, time of conversation with patients, administrative work, awkward working posture	9 (high)
Peros 2011 [43]	Croatia, Europe	dental school	dental students	152	152	current	back pain	95 (62.5)	not surveyed	8 (high)
Puriene 2008 [44]	Lithuania, Europe	dental practice, dental hospital/clinic	dentists	1,670	1,670	12 months	a) acute MSDs (general); b) chronic MSDs (general)	a) 1,445 (86.5); b) 651 (38.9)	not surveyed	7 (moderate)
Rising 2005 [28]	USA, North America	dental school	dental students	271	271	3 months	MSDs (general)	170 (62.7)	not surveyed	7 (moderate)
Rythkonen 2006 [52]	Finland, Europe	dental hospital/clinic	dentists	295	295	current	hand symptoms	142 (48.1)	working tasks (general)	8 (high)
Sakzewski 2015 [45]	Australia, Oceania	not described	dentists, orthodontists	466	466	7 days, 12 months	MSDs (general)	196 (42.0), 401 (86.0)	not surveyed	8 (high)
Samotoi 2008 [46]	New Zealand, Oceania	not described	dental therapists	323	323	7 days, 12 months	MSP (general)	see Table 3	not surveyed	7 (moderate)
Shaffer 2012 [24]	USA, North America	dental school	dental students	55	55	6 months	median mononeuro-pathy	6 (10.9)	not surveyed	7 (moderate)
Solovieva 2006 [29]	Finland, Europe	dental practice, dental hospital/clinic	dentists	291	291	10 years	Osteoarthritis a) general; b) thumb, index & middle fingers; c) ring & little fingers	a) 140 (48.1); b) 70 (24.0); c) 134 (46.0)	being dental professional, working tasks (general)	8 (high)
Sustova 2013 [47]	Czech Republic, Europe	not described	dentists	581	581	12 months	MSDs (general)	557 (95.8)	not surveyed	8 (high)
Thornton 2008 [48]	USA, North America	dental school, dental hospital/clinic	dental students	590	590	12 months	MSDs (general)	358 (60.6)	not surveyed	7 (moderate)
Vodanovic 2016 [17]	Croatia, Europe	dental practice	dentists	506	506	past years	MSP (general)	see Table 3	not surveyed	6 (moderate)
Warren 2010 [30]	USA, North America	dental school, dental hospital/clinic	dental hygienists, dental hygiene students	160	160	not stated	MSDs (general)	not stated	vibration, repetition, awkward working posture	8 (high)
Yee 2005 [49]	USA, North America	dental practice	dental hygienists	529	529	12 months	MSDs (general)	482 (91.1)	not surveyed	7 (moderate)
Zarra 2014 [58]	Greece, Europe	endodontic practice	endodontists	120	120	12 months	MSDs (general)	73 (60.8)	number of treated patients, awkward working posture	8 (high)
Zitzmann 2008 [50]	Switzerland, Europe	dental practice	dentists, dental hygienists, dental assistants	1,945	1,945	7 days	MSP (general)	see Table 3	not surveyed	7 (moderate)
Cohort studies (n = 3)										
Ding 2011 [26]	Finland, Europe	dental practice, schools	dentists, teachers	baseline: 543, follow up: 482	baseline: 295, follow up: 264	1 month at both surveys	osteoarthritis	baseline: 32 (10.8), 4 year follow up: not stated	not surveyed	10 (high)
Marklund 2008 [53]	Sweden, Europe	dental school	dental students	baseline: 371, follow up: 308	baseline: 371, follow up: 308	current at both surveys	a) jaw muscle symptoms; b) jaw muscle signs; c) myofascial pain	a) baseline: 41 (11.0), 1 year follow up: 47 (15.2); b) baseline: 129 (34.7), 1 year follow up: 138 (44.8); c) baseline: 25 (6.7), 1 year follow up: 24 (7.8)	not surveyed	8 (high)
Morse 2007 [54]	USA, North America	dental school, dental hospital/clinic	dental hygienists, dental hygiene students	160	160	12 months	MSP (general)	see Table 3	being dental professional, working tasks (general), awkward working posture	6 (moderate)
Case-control studies (n = 5)										
Cherniack 2008 [55]	USA, North America	not described	dental hygienists, shipyard workers	baseline: 308, follow up: 201	baseline: 94, follow up: 66	current at both surveys	CTS	baseline: 14 (14.9), 2 year follow up: 6 (9.0)	not surveyed	7 (moderate)
Ding 2010 [56]	Finland, Europe	not described	dentists, teachers	543	295	current	osteoarthritis	LH: 24 (8.1), RH: 32 (10.8)	dental instruments	8 (high)
Marklund 2010 [31]	Sweden, Europe	dental school	dental students	baseline: 280, follow ups: not stated	baseline: 280, follow ups: not stated	1 month, 12 months, 2 years	a) TMD symptoms; b) TMD signs; c) TMJ signs/ symptoms; d) jaw muscle signs/ symptoms	*a) baseline*: *70 (25*.*0);* *b) baseline*: *129 (46*.*0);* *c) baseline*: *84 (30*.*0)*, *1*^*st*^ *year follow-up*: *86 (12*.*0)*, *2*^*nd*^ *year follow-up*: *125 (28*.*0);* *d) baseline*: *104 (37*.*1)*, *1*^*st*^ *year follow-up*: *132 (27*.*0)*, *2*^*nd*^ *year follow-up*: *148 (26*.*0)*	not surveyed	10 (high)
Marklund 2010a [32]	Sweden, Europe	dental school	dental students	baseline: 280, follow ups: not stated	baseline: 280, follow ups: not stated	1 month, 12 months, 2 years	a) TMD symptoms; b) jaw pain; c) spinal pain; d) TMD pain	*a) 2*^*nd*^ *year follow-up*: *48 (17*.*1);* *b) 2*^*nd*^ *year follow-up*: *49 (17*.*5);* *c) 2*^*nd*^ *year follow-up*: *63 (22*.*5);* *d) 2*^*nd*^ *year follow-up*: *33 (11*.*7)*	not surveyed	10 (high)
Werner 2005 [15]	USA, North America	dental school, agencies	dental students, dental hygiene students, clerical workers	507	343	12 months	a) CTS; b) shoulder tendinitis; c) elbow tendinitis; d) wrist/hand/ finger tendinitis	a) 2 (0.58); b) 8 (2.33); c) 1 (0.29); d) 5 (1.45)	not surveyed	7 (moderate)

Abbreviations: CTS: Carpal Tunnel Syndrome, LH: left hand, MSDs/MSP: musculoskeletal diseases/musculoskeletal pain, RH: right hand, TMD: temporomandibular disorders, TMJ: temporomandibular joint, USA: United States of America

^a^ If the period of time is not stated in the study, it is labeled as “current” in this review.

^b^ If written in italic letters, incidence of MSDs/MSP was reported only.

^c^ If “see Table 3”, prevalence of MSDs/MSP was reported for body regions only.

A variety of dental professionals represented the sample in the included studies, for instance: dentists, orthodontists, dental hygienists, dental nurses, dental therapists, and dental students. Dentists were the most common study participants (n = 20, 48.7%), followed by dental students (n = 15, 36.5%), and dental hygienists (n = 11, 26.8%). The sample size ranged from 55 to 1,945 subjects, with an average of 448 subjects. Twenty-five studies (60.9%) analyzed musculoskeletal diseases or pain in general and 16 studies (39.1%) focused on a certain disease or kind of pain (e.g., CTS, osteoarthritis, back pain, TMJ pain). The most frequently used survey instrument was questionnaires (n = 24, 58.7%) [7,16,17,23,27, 28,33–50], followed by clinical examinations (n = 15, 36.5%) [14,15,24–26,29–32,51–56], posture analyses (n = 1, 2.4%) [57], and interviews (n = 1, 2.4%) [58].

Prevalence rates of musculoskeletal diseases and pain among dental professionals varied from 10.8% to 97.9%. The highest prevalence rate (97.9%) was reported in a high quality study for dentists suffering from musculoskeletal diseases in general within the previous 12 months [36]. With respect to the prevalence period, the point prevalence of musculoskeletal diseases and pain ranged from 11.0% to 95.9%, with a pooled prevalence rate of 58.0% (95% CI = 38.8–77.2, I^2^ = 98.8%, n = 12). The annual prevalence varied from 12.5% to 97.9%, with a pooled prevalence rate of 78.0% (95% CI = 60.2–95.8, I^2^ = 98.1%, n = 10).

Eight studies [16,24–26,29,51,55,56] examined the prevalence of particular musculoskeletal diseases among dental professionals. They have been diagnosed by clinical examinations including neuromuscular examinations, provocation and nerve conduction tests, vibration sensory tests, or x-rays depending on the analyzed study outcome. The prevalence of CTS among dental professionals ranged from 12.5% to 22.8% (n = 3). Prevalence rates of osteoarthritis varied from 10.8% to 48.1% (n = 4). One study reported the prevalence of median mononeuropathy (10.9%).

Based on the ten quality criteria, 20 studies (48.7%) were classified as high quality (8–10 points) [16,23,26,29–32,34,36,40–43,45,47,51–53,56,58] and 21 studies (51.3%) as moderate quality (5–7 points) [7,14,15,17,24,25,27,28,33,35,37–39,44,46,48–50,54,55,57], with an average of 7.5 points. Due to the third filter criterion, there were no studies of low quality. The most common reasons for a moderate methodological quality were weaknesses in study design, data analysis and response rate.

### Prevalence by body region

Several studies examined the prevalence in selected regions of the body (**Table 3**). In this review, the 10 most frequently analyzed body regions are displayed in descending order. The neck was the most frequently affected body region (in 15 out of 23 studies) [7,16,23,24,34–36,40,42,45–50], followed by the back (in 5 out of 23 studies) [14,17,38,42,44] and the shoulders (in 3 out of 23 studies) [37,45,54]. The annual prevalence of neck pain was between 29.1% and 84.8%. Back pain in general showed annual prevalence rates from 26.7% to 57.1%. That of lower back pain was higher and ranged from 28.5% to 74.9%. Shoulder pain had annual prevalence rates between 6.1% and 69.6%. The weekly prevalence of neck pain varied from 19.7% to 75.0%, that of lower back pain from 21.0% to 28.5% and that of shoulder pain from 16.7% to 28.9%. The point prevalence of neck pain was between 33.1% and 49.5%. Lower back pain showed point prevalence rates from 29.3% to 70.0%, and shoulder pain from 20.0% to 34.6%.

**Table 3:** Selected studies analyzing the prevalence of MSDs/MSP among dental professionals stratified by body region and period of time (n = 23).

Reference	Dental profes-sionals (n)	Prevalence: period of time^a^	Back^b^ (n+, %)	Neck (n+, %)	Hand/wrist (n+, %)	Shoulder (n+, %)	Elbow (n+, %)	Hip (n+, %)	Knee (n+, %)	Foot/ankle (n+, %)	Arm (n+, %)	Leg (n+, %)
Ayers 2009 [7]	566	7 days, 12 months	UB: 70 (12.3), 169 (29.8), LB: 120 (21.2), 325 (57.4)	112 (19.7), 332 (58.6)	56 (10.0), 141 (24.9)	95 (16.7), 257 (45.4)	23 (4.0), 57 (10.0)	30 (5.3), 84 (14.8)	54 (9.5), 118 (20.8)	34 (6.0), 74 (13.0)	not stated	not stated
Gijbels 2006 [14]	388	current	LB: 209 (53.8)	not stated	not stated	not stated	not stated	not stated	not stated	not stated	not stated	not stated
Harutunian 2011 [23]	74	6 months	30 (40.5), LB: 39 (52.7)	43 (58.1)	20 (27.0)	18 (24.3)	not stated	not stated	not stated	not stated	not stated	not stated
Hayes 2009a [34]	126	12 months	UB: 52 (41.2), LB: 72 (57.1)	81 (64.2)	52 (41.2)	60 (47.6)	7 (5.5)	14 (11.1)	33 (26.1)	16 (12.6)	8 (6.3)	4 (3.1)
Hayes 2013 [35]	560	12 months	UB: 346 (61.7), LB: 380 (67.8)	475 (84.8)	336 (60.0)	390 (69.6)	77 (13.7)	92 (16.4)	58 (10.3)	56 (10.0)	122 (21.7)	31 (5.5)
Hodacova 2015 [36]	575	12 months	UB: 286 (49.7), LB: 431 (74.9)	449 (78.0)	223 (38.7)	300 (52.1)	163 (28.3)	231 (40.1)	217 (37.7)	not stated	not stated	not stated
Humann 2015 [37]	488	current	UB: 145 (29.7), LB: 143 (29.3)	162 (33.1)	101 (20.6)	169 (34.6)	not stated	84 (17.2)	not stated	not stated	94 (19.2)	not stated
Kierklo 2011 [38]	220	current	44 (20.0), LB: 154 (70.0)	103 (46.8)	104 (47.2)	44 (20.0)	33 (15.0)	not stated	35 (15.9)	34 (15.4)	not stated	not stated
Leggat 2006 [40]	285	12 months	UB: 98 (34.3), LB: 153 (53.6)	164 (57.5)	96 (33.6)	152 (53.3)	37 (12.9)	36 (12.6)	54 (18.9)	33 (11.5)	not stated	not stated
Morse 2007 [54]	160	12 months	not stated	not stated	not stated	43 (26.8)	not stated	not stated	not stated	not stated	not stated	not stated
Palliser 2005 [42]	413	7 days, 12 months	UB: 72 (17.4), 133 (32.2), LB: 118 (28.5), 260 (62.9)	111 (26.8), 260 (62.9)	86 (20.8), 172 (41.6)	101 (24.4), 202 (48.9)	34 (8.2), 69 (16.7)	39 (9.4), 88 (21.3)	47 (11.3), 90 (21.7)	37 (8.9), 73 (17.6)	not stated	not stated
Pejcic 2017 [16]	356	current	LB: 163 (45.7)	176 (49.5)	not stated	not stated	not stated	not stated	not stated	not stated	not stated	not stated
Puriene 2008 [44]	1,670	12 months	acute: 1,520 (91.0), chronic: 954 (57.1)	not stated	acute: 1,388 (83.1), chronic: 508 (30.4)	not stated	not stated	not stated	not stated	not stated	not stated	not stated
Sakzewski 2015 [45]	466	7 days, 12 months	UB: 90 (19.3), 104 (22.3), LB: 130 (27.8), 133 (28.5)	130 (27.8), 143 (30.6)	68 (14.5), 78 (16.7)	135 (28.9), 143 (30.6)	35 (7.5), 39 (8.3)	55 (11.8), 60 (12.8)	not stated	not stated	not stated	not stated
Samotoi 2008 [46]	323	7 days, 12 months	UB: 47 (14.5), 98 (30.3), LB: 68 (21.0), 167 (51.7)	84 (26.0), 179 (55.4)	76 (23.5), 149 (46.1)	85 (26.3), 164 (50.7)	31 (9.5), 72 (22.2)	40 (12.3), 74 (22.9)	25 (7.7), 54 (16.7)	not stated	not stated	not stated
Shaffer 2012 [24]	55	6 months	not stated	15 (27.2)	3 (5.4)	not stated	not stated	not stated	not stated	not stated	15 (27.2)	not stated
Sustova 2013 [47]	581	12 months	UB: 286 (49.2), LB: 431 (74.1)	449 (77.2)	223 (38.3)	300 (51.6)	163 (28.0)	231 (39.7)	217 (37.3)	not stated	not stated	204 (35.1)
Thornton 2008 [48]	590	12 months	158 (26.7)	172 (29.1)	72 (12.2)	111 (18.8)	not stated	not stated	not stated	not stated	not stated	not stated
Vodanovic 2016 [17]	506	past years	UB: 386 (76.2), LB: 379 (74.9)	not stated	372 (73.5)		not stated	not stated	not stated	not stated	not stated	276 (54.5)
Werner 2005 [15]	343	12 months	not stated	not stated	69 (20.1)	21 (6.1)	55 (16.0)	not stated	not stated	not stated	see elbow	not stated
Yee 2005 [49]	529	12 months	UB: 323 (61.0), LB: 331 (62.5)	395 (74.6)	354 (66.9)	321 (60.6)	154 (29.1)	not stated	not stated	not stated	not stated	not stated
Zarra 2014 [58]	120	12 months	LB: 36 (30.0)	36 (30.0)	not stated	not stated	not stated	not stated	not stated	not stated	64 (53.3)	13 (10.8)
Zitzmann 2008 [50]	1,945	7 days	1,400 (71.9)	1,459 (75.0)	not stated	not stated	not stated	not stated	not stated	not stated	not stated	not stated

Abbreviations: LB: lower back, UB: upper back

^a^ If the period of time is not stated in the study, it is labeled as “current” in this review.

^b^ If no explanation is given, the prevalence belongs to the back in general.

Our meta-analysis showed consistent results for all prevalence periods, except point prevalence (**Table 4**). The greatest annual prevalence was observed for the neck 58.5% (95% CI = 46.0–71.0), the lower back 56.4% (95% CI = 46.1–66.8), the shoulders 43.1% (95% CI = 30.7–55.5), and the upper back 41.1% (95% CI = 32.3–49.9). The weekly prevalence was considerably lower: 35.1% (95% CI = 12.7–57.5), 24.5% (95% CI = 20.5–28.5), 23.9% (95% CI = 18.0–29.8), and 15.7% (95% CI = 12.5–18.9), respectively. In contrast to this, the highest point prevalence was indicated for the lower back 49.2% (95% CI = 33.0–65.4), the neck 42.8% (95% CI = 31.5–54.0), the hand/wrist 33.6% (95% CI = 7.6–59.6), and the shoulders 27.3% (95% CI = 13.0–41.7). For all examined body regions and periods of time, significant heterogeneity was observed between the included studies (I^2^ 22.3–99.2%). Heterogeneity was particularly high (> 84%) in 21 (out of 27) cases.

**Table 4:** Pooled prevalence rates of MSDs/MSP among dental professionals stratified by body region and period of time.

Prevalence: period of time^a^^,^^b^	Back (%, CI, I^2^^c^, n)	Upper back (%, CI, I^2^^c^, n)	Lower back (%, CI, I^2^^c^, n)	Neck (%, CI, I^2^^c^, n)	Hand/wrist (%, CI, I^2^^c^, n)	Shoulder (%, CI, I^2^^c^, n)	Elbow (%, CI, I^2^^c^, n)	Hip (%, CI, I^2^^c^, n)	Knee (%, CI, I^2^^c^, n)	Foot/ankle (%, CI, I^2^^c^, n)	Arm (%, CI, I^2^^c^, n)	Leg (%, CI, I^2^^c^, n)
Current	n/a	n/a	**49.2** (33.0–65.4) 95.2 (n = 4)	**42.8** (31.5–54.0) 87.0 (n = 3)	**33.6** (7.6–59.6) 96.3 (n = 2)	**27.3** (13.0–41.7) 92.4 (n = 2)	n/a	n/a	n/a	n/a	n/a	n/a
7 days	n/a	**15.7** (12.5–18.9) 66.7 (n = 4)	**24.5** (20.5–28.5) 66.5 (n = 4)	**35.1** (12.7–57.5) 99.2 (n = 5)	**16.9** (10.8–22.9) 90.4 (n = 4)	**23.9** (18.0–29.8) 85.1 (n = 4)	**7.1** (4.5–9.7) 77.5 (n = 4)	**9.5** (5.9–13.1) 84.4 (n = 4)	**9.4** (7.5–11.4) 22.3 (n = 3)	**7.2** (4.4–10.1) 62.9 (n = 2)	n/a	n/a
12 months	**41.9** (12.2–71.7) 99.1 (n = 2)	**41.1** (32.3–49.9) 95.5 (n = 10)	**56.4** (46.1–66.8) 95.5 (n = 11)	**58.5** (46.0–71.0) 97.3 (n = 12)	**35.9** (27.8–44.0) 97.4 (n = 13)	**43.1** (30.7–55.5) 98.4 (n = 13)	**17.2** (12.5–21.9) 94.3 (n = 11)	**21.2** (14.8–27.6) 95.2 (n = 9)	**23.6** (16.3–30.8) 95.3 (n = 8)	**12.8** (10.1–15.4) 60.3 (n = 5)	**25.7** (8.4–43.1) 96.5 (n = 3)	**13.5** (1.7–25.3) 97.8 (n = 4)

Abbreviations: CI: confidence interval, n/a: not applicable

^a^ If the period of time is not stated in the study, it is labeled as “current” in this review.

^b^ No pooled prevalence rates were calculated for the “6 months” or “past years” prevalence periods due to low use in the original studies.

^c^ I^2^ statistics: ≥ 25% considered low, ≥ 50% moderate and ≥ 75% high heterogeneity.

### Occupational risk factors

In the literature, a number of possible occupational risk factors for musculoskeletal diseases and pain among dental professionals have been identified. Findings of selected studies are displayed in **Table 5**. A review of the included studies revealed 15 occupational risk factors.

**Table 5:** Selected studies analyzing occupational risk factors for MSDs/MSP among dental professionals (n = 11).

Reference	Risk factor (predictor)^a^	MSDs/MSP (outcome)^b^	OR	95%-CI	p-value
***Profession***					
*1. Being dental professional*					
Morse 2007 [54]	being dental hygienist (ref. (non-) dental students)	neck pain	3.50	1.80–6.90*	<0.05
		shoulder pain	2.70	1.20–5.90*	<0.05
		multivariate analysis:			
		neck pain	5.00	1.70–15.00*	<0.05
		shoulder pain	2.70	1.20–5.90*	<0.05
Solovieva 2006 [29]	being dental specialist (ref. general dental practitioners)	osteoarthritis in any finger joint	1.22	0.63–2.35	ns
***Workplace***					
*2. Workplace*					
Hayes 2012 [27]	general private practice (ref. other practices)	shoulder pain	1.53	1.00–2.34*	<0.05
Hayes 2012 [27]	periodontal practice (ref. other practices)	forearm pain	2.42	1.19–4.90**	<0.01
***Work schedule***					
*3. Working time (hours/days)*					
Pejcic 2017 [16]	number of working days/week	MSP	0.60	0.47–0.77***	<0.001
		multivariate analysis:			
		MSP	0.92	0.55–1.56	ns
*4. No breaks*					
Pejcic 2017 [16]	no break between interventions	MSP	6.89	3.80–12.50***	<0.001
		multivariate analysis:			
		MSP	6.51	2.58–16.41***	<0.001
*5. Workload/job demand*					
Hodacova 2015 [36]	psychologically demanding work	low back pain	1.89	1.23–2.88**	<0.01
		neck pain	2.90	1.83–4.59**	<0.01
		shoulder pain	1.83	1.12–3.01*	<0.05
		multivariate analysis:			
		neck pain	2.39	1.46–3.89**	<0.01
***Patients***					
*6. Number of treated patients*					
Hodacova 2015 [36]	>20 patients/day (ref. <20 patients)	low back pain	1.56	1.11–2.20*	<0.05
		neck pain	1.36	1.00–1.92*	<0.05
		shoulder pain	1.50	1.03–2.20*	<0.05
Pejcic 2017 [16]	average number of patients	MSP	0.95	0.93–0.97***	<0.001
		multivariate analysis:			
		MSP	0.96	0.94–0.98***	<0.001
Zarra 2014 [58]	6–8 patients/day (ref. <6 patients)	MSDs	3.52	1.68–18.10*	<0.05
*7. Time of conversation with patients*					
Pejcic 2017 [16]	time of conversation with patients	MSP	3.16	1.45–6.89***	<0.001
		multivariate analysis:			
		MSP	2.13	0.52–8.71	ns
***Working tasks***					
*8. Working tasks (general)*					
Morse 2007 [54]	number of hours of cleaning teeth	neck pain	2.10	1.20–3.90*	<0.05
Morse 2007 [54]	amount of polishing teeth	shoulder pain	2.50	1.40–4.50*	<0.05
Rythkonen 2006 [52]	total time during work history in dental filling and root treatment	finger symptoms			
	(medium)		1.46	0.80–2.68	ns
	(high)		1.92	1.03–3.60*	<0.05
	(ref. low)				
Rythkonen 2006 [52]	average hours/week spent on dental filling and root treatment during the past 12 months	finger symptoms			
	(medium)		1.50	0.83–2.71	ns
	(high)		1.06	0.56–2.00	ns
	(ref. low)				
Solovieva 2006 [29]	cluster 2: 50% restorative treatments and endodontics, 50% prosthodontics, periodontics and surgical treatments	osteoarthritis in any finger joint	1.68	0.81–3.46	ns
	cluster 3: 100% restorative treatments and endodontics		1.59	0.86–2.93	ns
	(ref. cluster 1: variable working tasks)				
*9. Administrative work*					
Hayes 2009a [34]	computer-based work (<5 hours)	low back pain	16.83	2.44–138.13**	<0.01
		neck pain	12.89	1.92–102.69**	= 0.01
	(6–10 hours)	shoulder pain	7.03	1.42–39.49*	<0.05
		upper back pain	5.29	1.21–25.56*	<0.05
Hayes 2009a [34]	desk-based work (16–20 hours) (ref. <5 hours)	neck pain	19.70	1.34–378.94*	<0.05
Pejcic 2017 [16]	computer-based work	MSP	1.59	1.02–2.48*	<0.05
		multivariate analysis:			
		MSP	1.28	0.66–2.49	ns
*10. Hand scaling*					
Hayes 2012 [27]	hand scaling	neck pain	4.22	1.26–14.09*	<0.05
*11. Sonic/ultrasonic scaling*					
Hayes 2012 [27]	ultrasonic scaling	shoulder pain	3.11	1.20–8.05*	<0.05
		upper back pain	3.43	1.24–9.44*	<0.05
		lower back pain	2.76	1.07–7.13*	<0.05
***Work equipment***					
*12. Dental instruments*					
Ding 2010 [56]	fingers with low pinch strength	symptomatic osteoarthritis			
		(left hand)	2.00	1.10–3.80*	<0.05
		(right hand)	3.30	1.80–6.20*	<0.05
***Working conditions***					
*13. Vibration*					
Cherniack 2006 [51]	vibration exposure (years)	weak hand grip	1.77	1.12–2.80*	<0.05
	vibration exposure (years), raised VPT		1.55	1.14–2.12*	<0.05
Warren 2010 [30]	vibration (hours/day)	cold hands	1.03	not stated	ns
		numb/tingling	1.08	not stated	<0.10
		fingers numb/tingling & cold	1.05	not stated	ns
		neck symptoms	1.05	not stated	ns
		shoulder symptoms	0.97	not stated	ns
		elbow symptoms	1.09	not stated	<0.10
		forearm symptoms	1.12	not stated	ns
		back symptoms	0.96	not stated	ns
		decrease in hand grip strength	1.12	not stated	<0.05
		CTS right	1.13	not stated	<0.05
		CTS left	1.10	not stated	ns
		DeQuervains right	0.93	not stated	ns
		ulnar neuritis right	1.10	not stated	ns
		ulnar neuritis left	1.14	not stated	ns
		flexor tendinitis right	1.00	not stated	ns
		flexor tendinitis left	1.01	not stated	ns
		extensor tendinitis right	1.07	not stated	ns
		extensor tendinitis left	1.07	not stated	<0.10
*14. Repetition*					
Warren 2010 [30]	repetitive movements	cold hands	2.16	not stated	ns
		numb/tingling	2.21	not stated	<0.05
		fingers numb/tingling & cold	1.79	not stated	ns
		neck symptoms	1.42	not stated	ns
		shoulder symptoms	0.77	not stated	ns
		elbow symptoms	2.70	not stated	<0.10
		forearm symptoms	2.20	not stated	ns
		back symptoms	1.36	not stated	ns
		decrease in hand grip strength	2.80	not stated	<0.05
		CTS right	1.65	not stated	ns
		CTS left	2.46	not stated	ns
		DeQuervains right	439.51	not stated	ns
		ulnar neuritis right	0.87	not stated	ns
		ulnar neuritis left	0.73	not stated	ns
		flexor tendinitis right	7.94	not stated	<0.05
		flexor tendinitis left	2.54	not stated	ns
		extensor tendinitis right	1.77	not stated	<0.10
		extensor tendinitis left	1.42	not stated	ns
*15. Awkward working posture*					
Morse 2007 [54]	working with a bent neck	neck pain	2.10	1.30–3.40*	<0.05
Morse 2007 [54]	holding arms above shoulder height	shoulder pain	1.50	1.00–2.40*	<0.05
Pejcic 2017 [16]	working in the same position longer than 40 minutes	MSP	2.65	1.43–4.89**	<0.01
		multivariate analysis:			
		MSP	2.51	1.21–5.17*	<0.05
Pejcic 2017 [16]	discomfort while working in a certain body position	MSP	11.88	6.39–22.09***	<0.001
		multivariate analysis:			
		MSP	10.82	5.38–21.78***	<0.001
Pejcic 2017 [16]	preferred working position (sitting or standing)	MSP	1.63	1.11–2.38*	<0.05
		multivariate analysis:			
		MSP	2.02	1.20–3.42**	<0.01
Warren 2010 [30]	static reach/grip	cold hands	0.53	not stated	ns
		numb/tingling	1.02	not stated	ns
		fingers numb/tingling & cold	0.75	not stated	ns
		neck symptoms	0.83	not stated	ns
		shoulder symptoms	1.20	not stated	ns
		elbow symptoms	0.69	not stated	ns
		forearm symptoms	0.67	not stated	ns
		back symptoms	0.90	not stated	ns
		decrease in hand grip strength	1.48	not stated	ns
		CTS right	1.25	not stated	ns
		CTS left	1.97	not stated	ns
		DeQuervains right	1.03	not stated	ns
		ulnar neuritis right	2.00	not stated	ns
		ulnar neuritis left	1.82	not stated	ns
		flexor tendinitis right	0.96	not stated	ns
		flexor tendinitis left	1.05	not stated	ns
		extensor tendinitis right	0.70	not stated	ns
		extensor tendinitis left	0.95	not stated	ns
Zarra 2014 [58]	awkward postures during clinical practice (ref. no awkward postures)	MSDs	4.56	1.34–15.51*	<0.05

Abbreviations: CI: confidence interval, CTS: carpal tunnel syndrome, MSDs/MSP: musculoskeletal diseases/musculoskeletal pain, ns: not significant (p>0.05), OR: odds ratio, ref: reference group, VPT: vibrotactile perception thresholds

^a^ If no reference group is given, the information is not stated in the study.

^b^ If a uni- and multivariate regression analysis was conducted, the first results refer to the univariate and the following to the multivariate analysis.

* significant result

** very significant result

*** highly significant result.

The prevalence of musculoskeletal pain seems to be positively associated with being a dental professional. One study found significantly higher odds of suffering from neck or shoulder pain for dental hygienists compared to (non-) dental students (AOR = 5.00, CI = 1.70–15.00, p < 0.05; AOR = 2.70, CI = 1.20–5.90, p < 0.05) [54]. Pejcic et al. reported that having no breaks between interventions significantly increased the odds of musculoskeletal pain (AOR = 6.51, CI = 2.58–16.41, p < 0.001) [16]. Therefore, the work schedule appears to have an important influence on the prevalence of musculoskeletal diseases and pain. Beyond this, another study stated that psychologically demanding work probably caused neck pain among dental professionals (AOR = 2.39, CI = 1.46–3.89, p < 0.01) [36]. Zarra et al. examined the influence of the number of treated patients on the risk of suffering from musculoskeletal diseases. They found that the odds were 3.52 times higher for dental professionals who treated 6 to 8 patients per day in comparison to colleagues treating less than 6 patients (CI = 1.68–18.10, p < 0.05) [58]. Hodacova et al. confirmed this result but for 20 patients per day [36].

In addition, several working tasks appear to be positively correlated with the risk of musculoskeletal pain. One study indicated that an increasing number of hours of cleaning teeth increased the odds of neck pain (OR = 2.10, CI = 1.20–3.90, p < 0.05) [54]. The authors also found that the amount of teeth polishing done is a possible etiological factor for shoulder pain (OR = 2.50, CI = 1.40–4.50, p < 0.05) [54]. Hayes et al. described that hand scaling significantly increased the odds of neck pain (OR = 4.22, CI = 1.26–14.09, p < 0.05) and ultrasonic scaling the odds of shoulder pain (OR = 3.11, CI = 1.20–8.05, p < 0.05), upper back pain (OR = 3.43, CI = 1.24–9.44, p < 0.05), and lower back pain (OR = 2.76, CI = 1.07–7.13, p < 0.05) [27]. Furthermore, the authors stated in a previous study that musculoskeletal pain may have been caused by administrative work. Dental professionals who performed desk-based work for 16–20 hours ran a 19.70 times higher odds of having neck pain than colleagues performing fewer than 5 hours (CI = 1.34–378.94, p < 0.05) [34]. Similar results were reported for computer-based work [34]. One study investigated the influence of vibration on the risk of musculoskeletal diseases, such as CTS. The results indicated that vibration increased the odds of CTS in the right hand by 1.13 times (CIs not stated, p < 0.05) [30].

Moreover, awkward working posture through cramped, twisted and prolonged sitting or standing positions was the most frequently analyzed etiological factor of musculoskeletal diseases and pain. Pejcic et al. demonstrated that discomfort while working in a certain body position probably caused musculoskeletal pain among dental professionals (AOR = 10.82, CI = 5.38–21.78, p < 0.001). The authors also showed that working in the same position longer than 40 minutes significantly increased the odds of musculoskeletal pain (AOR = 2.51, CI = 1.21–5.17, p < 0.05) [16]. These findings were in line with conclusions of other studies [30,54]. Finally, in a study by Zarra et al., dental professionals who worked with awkward postures during clinical practice ran a 4.56 times higher odds of musculoskeletal diseases than colleagues working without awkward postures (CI = 1.34–15.51, p < 0.05) [58].

## Discussion

This literature review presents the most current state of research on the prevalence and occupational risk factors of musculoskeletal diseases and pain among dental professionals in Western countries. Our findings were drawn from 41 research articles published from 2005 to 2017. Prevalence rates of musculoskeletal diseases and pain ranged from 10.8% to 97.9%, with a pooled annual prevalence rate of 78.0% (95% CI = 60.2–95.8). In most of the studies, the prevalence rates were high (above 60%). Therefore, dental professionals are particularly at risk of musculoskeletal diseases and pain.

This was also evident when looking at the prevalence rates of the individual body regions. In this review and meta-analysis, the neck was the most frequently affected body region (pooled annual prevalence: 58.5%, 95% CI = 46.0–71.0). Ohlendorf et al. conducted a kinematic analysis of occupational musculoskeletal loadings in several body regions among dentists. The authors observed disadvantageous joint angle distributions in the 75th and 95th percentile of the head and cervical spine during the treatment of patients. This static working posture possibly contributed to particular muscular strains in the neck [59,60]. Some other studies also found high prevalence rates for neck pain (above 66%) among Asian dental professionals [2,61,62]. In the back region, lower back pain (pooled annual prevalence: 56.4%, 95% CI = 46.1–66.8) was most common compared to the back in general (pooled annual prevalence: n/a) and the upper back (pooled annual prevalence: 41.1%, 95% CI = 32.3–49.9). One study observed among dental professionals a characteristic twisting of the back during the treatment of patients, caused by a greater right tilt of the lumbar spine and a left tilt of the thoracic spine [59]. Howarth et al. found similar results in their study and concluded that forced postures while sitting are significantly associated with pain in the lower back. Dental hygienists, for instance, spend 66% of their working time seated, 40% of it with a forward bent trunk posture of 30 degrees [63]. High prevalence rates for lower back pain (above 57%) among Asian dental professionals were also reported by Aljanakh et al. [1], Batham et al. [61] and Kumar et al. [64]. Furthermore, our meta-analysis showed that musculoskeletal diseases and pain were also common in the shoulders (pooled annual prevalence: 43.1%, 95% CI = 30.7–55.5) and in the hand/wrist (pooled point prevalence: 33.6%, 95% CI = 7.6–59.6). Several other studies confirmed this result for Asian dental professionals [2,62,65,66]. These findings suggest that dental professionals primarily use upper body regions at work. Especially during the treatment of patients and administrative work, which account for around 70% of all dental tasks, upper extremities like the hand/wrist or shoulder are increasingly under muscular strain [59]. Thus, dental professionals are particularly vulnerable to musculoskeletal diseases and pain in the shoulder and hand/wrist.

This literature review revealed 15 possible occupational risk factors for musculoskeletal diseases and pain among dental professionals. Our findings indicate that administrative work like desk-based (OR = 19.70, CI = 1.34–378.94) and computer-based (OR = 12.89, CI = 1.92–102.69) work had the highest significant influence on the prevalence of musculoskeletal pain (here: neck pain) [34]. However, the large confidence intervals might indicate that these results are not robust. Several studies found that administrative work on the computer or desk is combined with disadvantageous static sitting positions that may cause twisting, forced postures and eventually diseases or pain in the neck or other body regions [16,30,54,63]. The second most important occupational risk factor was the awkward working posture. Pejcic et al. found that discomfort while working in a certain body position significantly increased the odds of musculoskeletal pain (AOR = 10.82, CI = 5.38–21.78) [16]. Several other studies from Asia and South America found similar results for dental professionals [64,67,68]. Awkward working postures result from specific dental tasks like hand scaling, ultrasonic scaling and cleaning/polishing teeth [27,54]. Consequently, dental professionals are particularly vulnerable to musculoskeletal diseases and pain due to forced postures. In third place, the work schedule seems to be associated with the risk of musculoskeletal diseases and pain. One study reported that having no breaks between interventions significantly increased the odds of musculoskeletal pain by 6.51 times (CI = 2.58–16.41) [16]. Fals Martínez et al. confirmed this finding for South American dental professionals [67]. Insufficient breaks during dental activities that are very demanding to the musculoskeletal system lead to an overstraining of the system. As a consequence, musculoskeletal diseases and pain can occur [16]. Finally, several dental tasks appear to be correlated with the prevalence of musculoskeletal diseases and pain. For instance, Hayes et al. stated that hand scaling may have caused neck pain among dental professionals (OR = 4.22, CI = 1.26–14.09) [27]. One other study from South Africa showed similar results [69]. Some dental tasks like hand scaling increase the risk of musculoskeletal diseases and pain especially due to awkward working postures that are combined with the performance of this dental activity (see above) [27,54].

Ultimately, our findings indicate that many occupational risk factors are associated with musculoskeletal diseases and pain among dental professionals. However, it is evident that the individual risk factors were analyzed in only a few studies. Therefore, it was difficult to compare results for the individual risk factors. Furthermore, musculoskeletal diseases and pain are multifactorial [70]. Often, several factors play a role in the development of musculoskeletal diseases and pain, and the respective degree of influence is difficult to determine. Kihun et al. stated that musculoskeletal diseases and pain are influenced by physical, occupational, and socio psychological factors (see background) [8]. Hence, occupational risk factors are one of a number of factors contributing to the causation of musculoskeletal diseases and pain [70]. However, the findings of the current review showed that an occupation in dentistry and its related factors significantly contribute to the development of musculoskeletal diseases and pain.

Moreover, several intervention studies already examined the effectiveness of various preventative measures against musculoskeletal diseases and pain among dental professionals [43,71–73]. The studies found, for instance, that regular physical activity before and after work, back exercises, dynamic sitting, and magnification loupes can significantly contribute to the reduction of musculoskeletal diseases and pain among dental professionals.

### Strengths and limitations

This literature review and meta-analysis as well as its included studies contain several methodological strengths and limitations. Firstly, this work only considered studies from Western countries. Therefore, the working conditions and environment of the individual oral healthcare facilities in the studies can be considered as similar. As a consequence, the study results were comparable with each other when focusing on the geographical areas.

Furthermore, the literature search revealed many studies related to the described research topic. However, the research focus of the included sources often varied from each other. The studies used different study designs and outcomes related to musculoskeletal diseases and pain, diverging subgroups according to the study population and different periods of time related to the prevalence of musculoskeletal diseases and pain. The comparability of the study results was limited by this reason so that general conclusions were difficult to make. This weakness was solved by summarizing and pooling the results by topic (e.g., separately for the prevalence period, outcomes, and body regions).

In addition, the included studies used divergent survey instruments like a questionnaire (58.7%) (mainly a modified version of the Standardized Nordic Questionnaire [74]), clinical examination (36.5%) and posture analysis or interview (both 2.4%). The Nordic Questionnaire is a well-validated and common measurement tool that seems to be suitable for examining the prevalence of musculoskeletal diseases and pain [74]. In all cases, the clinical examination, posture analysis, and interview were conducted using standardized well-validated checklists or other survey instruments. Consequently, the results of the included studies were accurate and mutually comparable in this case. It was therefore possible to draw reliable conclusions and generalize the results if they were analyzed and pooled by topic (see above).

The current research topic showed many studies (n = 945) that were suitable for inclusion in this review. Due to the high number of studies additional filter criteria were applied. Overall, 41 studies were included, which correspond to a sufficient number of studies representing relevant data for this review.

Further, this review only considered observational studies from peer reviewed journals and no grey literature. Therefore, a sufficient methodological quality of the studies was ensured. Moreover, there were many missing values in the original studies, but the majority of missing data could be calculated so that presented results only contain few missing values (see **Tables 2–5**).

The assessment of the methodological study quality was carried out with an instrument based on two standardized, well-validated checklists [18–20]. The instrument was applicable to all types of observational studies that were included. The study quality of included sources was satisfactory, with an average of 7.5 points. Almost half of the studies had a high (48.7%) or moderate (51.3%) quality. By employing the third filter criterion, there were no studies of low quality. However, the quality assessment revealed some weaknesses in methodology. The majority of included studies (n = 37, 90.2%) did not use a prospective or retrospective study design for examining the cause effect relationship between occupational risk factors and musculoskeletal diseases and pain. Likewise, more than half of the studies (n = 25, 60.9%) did not control for confounding in their statistical analyses. The response rate was below 70% in around half of the studies (n = 22, 53.6%).

Our meta-analysis included 30 out of 41 studies that were 73.1%. Reasons for the exclusion of 11 studies were a differing prevalence period or missing prevalence data. The pooled analysis enabled comparability of prevalence data from different studies. The applied analysis tool from Neyeloff et al. [21] was an appropriate and simple option for pooling and weighting prevalence data from several studies. Moreover, significant and high heterogeneity between the included studies was observed, especially resulting from different study designs, study populations, survey instruments, and study outcomes. As a result of this and a small number of studies in the sub-analyses, the described pooled prevalence rates should be interpreted cautiously.

## Conclusions

This literature review showed evidence that musculoskeletal diseases and pain are a major health burden for dental professionals. Our findings revealed high prevalence rates for various diseases and types of pain related to the musculoskeletal system. Several body regions were affected by musculoskeletal diseases and pain, especially the neck, back, and shoulders. In addition, many possible occupational risk factors for musculoskeletal diseases and pain could be identified, such as awkward working posture, hand scaling and high number of treated patients. Hence, suitable interventions for preventing musculoskeletal diseases and pain among dental professionals are needed. In the long term this could significantly reduce the burden of disease, costs of illness, absenteeism from work, and occupational accidents.

The results of our study are a valuable base for occupational health practitioners or accident and health insurances. The stakeholders can employ our work for the development of promoting health and preventing interventions. Identified occupational risk factors of this review offer initial ideas for the development of preventing measures. A good ergonomic design of the dental workplace is especially essential to reduce awkward working postures during clinical practice and administrative work. Therefore, further research is needed on possible ergonomic and organizational measures to reduce musculoskeletal diseases and pain among dental professionals for the long term.

In sum, this review indicates that many studies in Western countries focused on the prevalence and occupational risk factors of musculoskeletal diseases and pain among dental professionals. But there is still no current study from Germany. More longitudinal studies examining the etiology of musculoskeletal diseases and pain among dental professionals are essential.

## Supporting information

S1 Appendix Detailed general search strategy for all included databases. (247 KB PDF).
(PDF)

S1 Table PRISMA checklist. (309 KB PDF).
(PDF)

## Abbreviations

AOR: adjusted odds ratio
BGW: Institution for Statutory Accident Insurance and Prevention in the Health and Welfare Services
CI: confidence interval
CTS: carpal tunnel syndrome
LB: lower back
LH: left hand
MSDs/MSP: musculoskeletal diseases/musculoskeletal pain
n/a: not applicable
ns: not significant
OR: odds ratio
PEOS: population exposure, outcome, study design
PRISMA: preferred reporting items for systematic reviews and meta-analyses
ref: reference group
RH: right hand
TMD: temporomandibular disorders
TMJ: temporomandibular joint
UB: upper back
UKE: University Medical Center Hamburg-Eppendorf
USA: United States of America
VPT: vibrotactile perception thresholds
