# Supplementary material for: Prevalence and occupational risk factors of musculoskeletal diseases and pain among dental professionals in Western countries: A systematic literature review and meta-analysis
Source: PLoS One. 2018 Dec 18;13(12):e0208628. doi: 10.1371/journal.pone.0208628 (PMC6298693; doi:10.1371/journal.pone.0208628)
Supplement: S1 Appendix — (247 KB PDF). (PDF) [file pone.0208628.s001.pdf]

S1_Appendix

1 Dent* (dental personnel)
2 Health Occupations
3 Oral Health
4 Orthodontists
5 Anaplastologists
6 Orthodontic Assistants
7 Laboratory Personnel
8 1 OR 2 OR 3 OR 4 OR 5 OR 6 OR 7
9 Occupational Diseases
10 Occupational Injuries
11 Occupational Accidents
12 Occupational Medicine/Occupational Exposure/Occupational Health
13 Occupational Risk*
14 Risk Factors
15 Work-related
16 Biomechanical*
17 Human Engineering
18 Rotation
19 Vibration
20 Physical Exertion
21 Repetition
22 Force
23 Hand Grip
24 High Work Pace
25 Posture
26 9 OR 10 OR 11 OR 12 OR 13 OR 14 OR 15 OR 16 OR 17 OR 18 OR 19 OR 20 OR 21 OR 22 OR 23 OR 24 OR 25
27 Musculoskeletal Diseases
28 Musculoskeletal Pain
29 Work-Related Upper-Extremity Musculoskeletal Disorders
30 Neck Pain
31 Shoulder Pain
32 Back Pain
33 Carpal Tunnel Syndrome
34 Cubital Tunnel Syndrome
35 Radial Tunnel Syndrome
36 Guyon Canal Syndrome
37 Thenar-Hammer-Syndrome
38 Median Mononeuropathy
39 Cervical Lordosis
40 Arthrosis
41 Tenosynovitis

42	Tendinopathy
43	27 OR 28 OR 29 OR 30 OR 31 OR 32 OR 33 OR 34 OR 35 OR 36 OR 37 OR 38 OR 39 OR 40 OR 41 OR 42
44	8 AND 26 AND 43
45	Limit 44 to yr="1980-Current"
46	Limit 45 to lang="English"
